# 4-(2-Nitro­benz­yl)-3-phenyl-3,4-di­hydro-2*H*-1,4-benzoxazin-2-ol

**DOI:** 10.1107/S1600536814015645

**Published:** 2014-07-11

**Authors:** Louisa Chouguiat, Raouf Boulcina, Sofiane Bouacida, Hocine Merazig, Abdelmadjid Debache

**Affiliations:** a Laboratoire de Synthèse des Molécules d’Intérêts Biologiques, Département de Chimie, Faculté des Sciences Exactes, Université de Constantine 1, 25000 Constantine, Algeria; b Unité de Recherche de Chimie de l’Environnement et Moléculaire Structurale, CHEMS, Université Constantine 1, 25000 , Algeria; c Département Sciences de la Matière, Faculté des Sciences Exactes et Sciences de la Nature et de la Vie, Université Oum El Bouaghi 04000, Algeria

**Keywords:** crystal structure

## Abstract

The title compound, C_21_H_18_N_2_O_4_, crystallizes with two independent mol­ecules (*A* and *B*) in the asymmetric unit. In both mol­ecules the oxazine ring has an envelope conformation with the hydroxyl-substituted C atom as the flap. The nitro­benzyl ring and the phenyl ring are almost normal to the mean plane of the benzooxazine ring system with dihdral angles of 85.72 (15) and 82.69 (15)°, respectively, in mol­ecule A, and 85.79 (15) and 87.72 (15)°, respectively, in mol­ecule B. The main difference in the conformation of the two mol­ecules concerns the dihedral angle between the nitro­benzyl ring and the phenyl ring, *viz.* 79.67 (18) in mol­ecule *A* and 71.13 (18)° in mol­ecule *B*. In the crystal, the *A* and *B* mol­ecules are linked by an O—H⋯O hydrogen bond. These units are then linked *via* C—H⋯O hydrogen bonds, forming sheets lying parallel to (010). Further C—H⋯O hydrogen bonds link the sheets to form a three-dimensional network. There are also O—H⋯π and C—H⋯π inter­actions present, reinforcing the three-dimensional structure.

## Related literature   

For the preparation and applications of similar structures, see: Ozden *et al.* (1992 ▶); Hartenstein & Sicker (1994 ▶); Ilas *et al.* (2005 ▶); Touzeau *et al.* (2003 ▶); Torisu *et al.* (2004 ▶); Largeron *et al.* (1999 ▶).
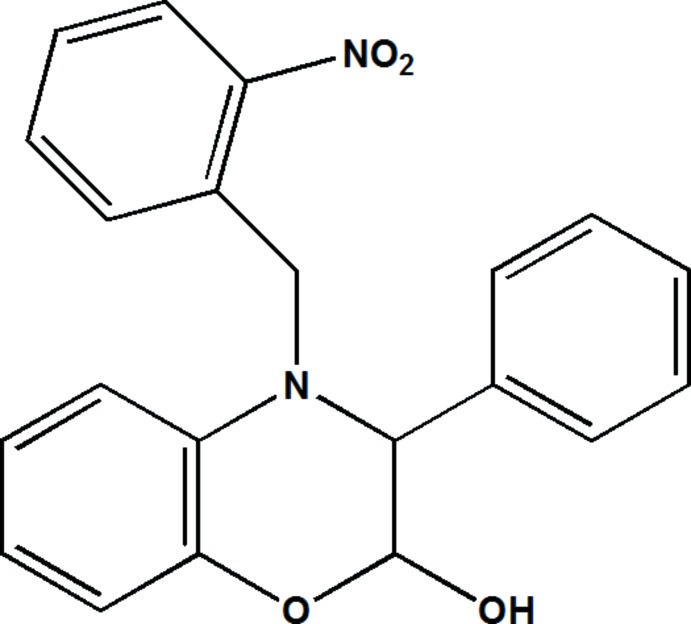


## Experimental   

### 

#### Crystal data  

C_21_H_18_N_2_O_4_
*M* *_r_* = 362.37
Orthorhombic, 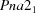

*a* = 12.7332 (14) Å
*b* = 14.2777 (14) Å
*c* = 19.003 (2) Å
*V* = 3454.8 (6) Å^3^
*Z* = 8
Mo *K*α radiation
μ = 0.10 mm^−1^
*T* = 150 K
0.13 × 0.05 × 0.03 mm

#### Data collection  

Bruker APEXII CCD area-detector diffractometer
Absorption correction: multi-scan (*SADABS*; Sheldrick, 2002 ▶) *T*_min_ = 0.860, *T*_max_ = 1.000
19156 measured reflections
3161 independent reflections
4907 reflections with *I* > 2σ(*I*)
*R*_int_ = 0.052

#### Refinement  

*R*[*F* ^2^ > 2σ(*F* ^2^)] = 0.043
*wR*(*F* ^2^) = 0.114
*S* = 1.04
3161 reflections
489 parameters
1 restraint
H-atom parameters constrained
Δρ_max_ = 0.71 e Å^−3^
Δρ_min_ = −0.26 e Å^−3^

### 

Data collection: *APEX2* (Bruker, 2011 ▶); cell refinement: *SAINT* (Bruker, 2011 ▶); data reduction: *SAINT*; program(s) used to solve structure: *SIR2002* (Burla *et al.*, 2003 ▶); program(s) used to refine structure: *SHELXL97* (Sheldrick, 2008 ▶); molecular graphics: *ORTEP-3 for Windows* (Farrugia, 2012 ▶) and *DIAMOND* (Brandenburg & Berndt, 2001 ▶); software used to prepare material for publication: *WinGX* publication routines (Farrugia, 2012 ▶).

## Supplementary Material

Crystal structure: contains datablock(s) I. DOI: 10.1107/S1600536814015645/bq2396sup1.cif

Structure factors: contains datablock(s) I. DOI: 10.1107/S1600536814015645/bq2396Isup2.hkl

Supporting information file. DOI: 10.1107/S1600536814015645/bq2396Isup3.cml

CCDC reference: 1012140

Additional supporting information:  crystallographic information; 3D view; checkCIF report

## Figures and Tables

**Table 1:** Hydrogen-bond geometry (Å, °) *Cg*1 and *Cg*2 are the controids of the C10*A*–C15*A* and C10*B*–C15*B* rings, respectively.

*D*—H⋯*A*	*D*—H	H⋯*A*	*D*⋯*A*	*D*—H⋯*A*
O2*A*—H2*A*⋯O1*B*	0.82	2.06	2.843 (4)	161
C6*B*—H6*B*⋯O21*A* ^i^	0.93	2.40	3.175 (5)	141
C8*B*—H8*B*⋯O1*A* ^ii^	0.98	2.34	3.234 (4)	151
C14*A*—H14*A*⋯O2*A* ^iii^	0.93	2.57	3.177 (5)	123
C19*A*—H19*A*⋯O21*B* ^iv^	0.93	2.45	3.134 (4)	131
C19*B*—H19*B*⋯O22*A* ^v^	0.93	2.47	3.057 (5)	121
O2*B*—H2*B*⋯*Cg*1^ii^	0.82	2.69	3.484 (3)	164
C18*A*—H18*A*⋯*Cg*2^iv^	0.93	2.83	3.564 (4)	137
C18*B*—H18*B*⋯*Cg*1^v^	0.93	2.94	3.601 (4)	130

Symmetry codes: (i) 
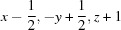
; (ii) 
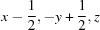
; (iii) 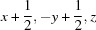; (iv) 
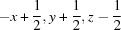
; (v) 
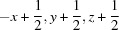
.

**Fig. 1.**
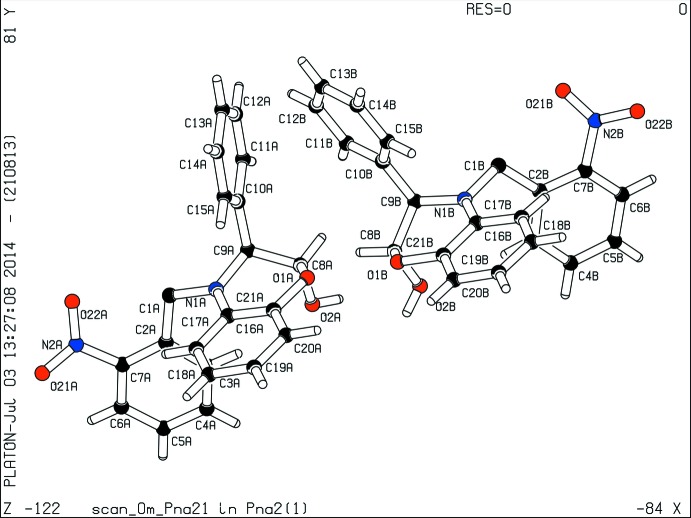
The title molecule (Farrugia, 2012) with the atomic labelling scheme. The displacement parameters are drawn at the 50% probability level.

**Fig. 2.**
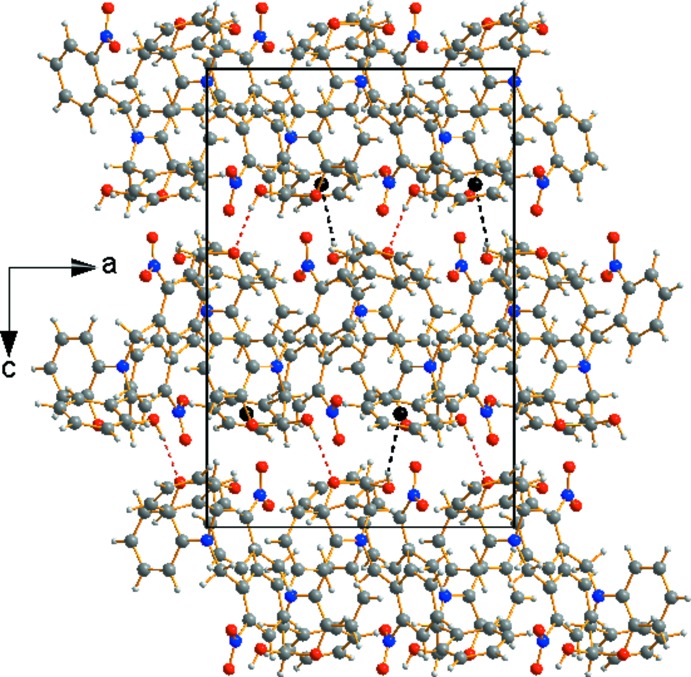
(Brandenburg & Berndt, 2001) Part of the crystal structure viewed down the b axis showing alternating layers and O—H···O hydrogene bond (in red)and O—H···π interactions.

## supplementary crystallographic information

### S1. Comment

Numerous natural and synthetic substances that have the core "1,4-benzoxazine" have been used in different fields of medicine. The 1,4-benzoxazine structure is an integral part of several naturally occurring substances. For example, various glycosides of the 2-hydroxy-2*H*-1,4-benzoxazine skeletons have been found to occur in gramineous plants such as maize, wheat, rye, and rice, and have been suggested to act as plant resistance factors against microbial diseases and insects (Ozden *et al.*, 1992; Hartenstein & Sicker, 1994). Moreover, 3,4-Dihydro-2*H*-1,4-benzoxazines have received a great deal of attention due to their wide range of biological and therapeutical properties (Ilas *et al.*, 2005). For example they have been investigated as antihypertensive agents (Touzeau *et al.*, 2003), neuroprotective antioxidants (Largeron *et al.*, 1999) and prostaglandin D 2 receptor antagonists (Torisu *et al.*, 2004). Herein, we report our results about the synthesis and the crystallographic study of 4-(2-nitrobenzyl)-3-phenyl-3,4-dihydro-2*H*-benzo[*b*][1,4]oxazin-2-ol, (I). The molecular geometry and the atom-numbering scheme of asymetric unit are shown in Fig. 1. The asymetric unit contents two molecule of (I). The crystal packing can be described as alternating connected layers parallel to the (001) plane along the *c* axis (Fig. 2) It is stabilized by intra et intermolecular O—H···O and C—H···O hydrogen bond and O—H···π interactions (Table 1; Fig. 2). These interactions link the molecules within the layers and also link the layers together and reinforcing the cohesion of the structure.

### S2. Experimental

A mixture of 4-methyl-2-(2-nitrobenzylamino)phenol (1 mmol), boronic acid (1 mmol) and glyoxal (1 mmol) in methanol (5 ml) was stirred at room temperature for 24 h. The solvent was removed *in vacuo* to give crude producs, which was purified by flash chromatography (silica gel, dichloromethane). Spectroscopic data for the major isomer are given below. IR (KBr): ν: 3429, 2920, 1608, 1512, 1250, 1036 cm-1. 1H NMR (250 MHz, CDCl3, J Hz) δ: 8.09 (d, 1H, J=7.5 Hz, CH arom); 7.91 (d, 1H, J=7.5 Hz, CH arom.); 7.56 (t, 1H, J=7.5 Hz, CH arom.); 7.42 (t, 1H, J=7.5 Hz, CH arom); 7.33–7.30 (m, 3H, CH arom.); 7.21–7.18 (m, 2H, CH arom.); 6.92–6.83 (m, 2H, H arom.); 6.74–6.67 (m, 1H, CH arom.); 6.45 (d, 1H, J= 7.5 Hz, CH arom.); 5.60 (s, 1H, H2); 5.06 (d, 1H, J= 18.5 Hz, Hb); 4.62 (d, 1H, J=18.5 Hz, Ha); 4.51 (s, 1H, H3); 3.26 (s, 1H, OH). 13 C NMR (62.9 MHz, CDCl3) δ: 148.1; 141.1; 139.7; 138.3; 134.1; 134.0; 133.7; 129.0; 130.0; 128.3; 128.0; 126.9; 125.3; 122.9; 117.9; 117.5; 110.6; 92.7; 64.3; 50.2. HRMS: (*M*+H)+, found 363.1347, C_21_H_19_N_2_O_4_ requires 363.1345.

### S3. Refinement

All H atoms were localized on Fourier maps but introduced in calculated positions and treated as riding on their parent atoms (C and O) with C—H = 0.97 Å (methylene); C—H = 0.93 Å (aromatic) or C—H = 0.98 Å (methine); O—H = 0.82 Å and with *U*_iso_(H) = 1.2 *U*_eq_(C_aryl_; C_methine_ or C_methylene_)and *U*_iso_(H) = 1.5 *U*_eq_(O_hydroxy_). In the absence of significant anomalous scattering effects Friedel pairs have been merged. The number of Friedel pairs is 2686.

### Crystal data

**Table:**

C_21_H_18_N_2_O_4_	*F*(000) = 1520
*M**_r_* = 362.37	*D*_x_ = 1.393 Mg m^−^^3^
Orthorhombic, *P**n**a*2_1_	Mo *K*α radiation, λ = 0.71073 Å
Hall symbol: P 2c -2n	Cell parameters from 4509 reflections
*a* = 12.7332 (14) Å	θ = 2.4–24.4°
*b* = 14.2777 (14) Å	µ = 0.10 mm^−^^1^
*c* = 19.003 (2) Å	*T* = 150 K
*V* = 3454.8 (6) Å^3^	Stick, colourless
*Z* = 8	0.13 × 0.05 × 0.03 mm

### Data collection

**Table:**

Bruker APEXII CCD area-detector diffractometer	4907 reflections with *I* > 2σ(*I*)
Graphite monochromator	*R*_int_ = 0.052
φ and ω scans	θ_max_ = 25.1°, θ_min_ = 2.9°
Absorption correction: multi-scan (*SADABS*; Sheldrick, 2002)	*h* = −15→14
*T*_min_ = 0.860, *T*_max_ = 1.000	*k* = −17→16
19156 measured reflections	*l* = −20→22
3161 independent reflections	

### Refinement

**Table:**

Refinement on *F*^2^	Primary atom site location: structure-invariant direct methods
Least-squares matrix: full	Secondary atom site location: difference Fourier map
*R*[*F*^2^ > 2σ(*F*^2^)] = 0.043	Hydrogen site location: inferred from neighbouring sites
*wR*(*F*^2^) = 0.114	H-atom parameters constrained
*S* = 1.04	*w* = 1/[σ^2^(*F*_o_^2^) + (0.0733*P*)^2^ + 1.1329*P*] where *P* = (*F*_o_^2^ + 2*F*_c_^2^)/3
3161 reflections	(Δ/σ)_max_ < 0.001
489 parameters	Δρ_max_ = 0.71 e Å^−^^3^
1 restraint	Δρ_min_ = −0.26 e Å^−^^3^

### Special details

**Table:**

Geometry. All e.s.d.'s (except the e.s.d. in the dihedral angle between two l.s. planes) are estimated using the full covariance matrix. The cell e.s.d.'s are taken into account individually in the estimation of e.s.d.'s in distances, angles and torsion angles; correlations between e.s.d.'s in cell parameters are only used when they are defined by crystal symmetry. An approximate (isotropic) treatment of cell e.s.d.'s is used for estimating e.s.d.'s involving l.s. planes.
Refinement. Refinement of *F*^2^ against ALL reflections. The weighted *R*-factor *wR* and goodness of fit *S* are based on *F*^2^, conventional *R*-factors *R* are based on *F*, with *F* set to zero for negative *F*^2^. The threshold expression of *F*^2^ > σ(*F*^2^) is used only for calculating *R*-factors(gt) *etc*. and is not relevant to the choice of reflections for refinement. *R*-factors based on *F*^2^ are statistically about twice as large as those based on *F*, and *R*- factors based on ALL data will be even larger.

### Fractional atomic coordinates and isotropic or equivalent isotropic displacement parameters (Å^2^)

**Table:**

	*x*	*y*	*z*	*U*_iso_*/*U*_eq_	
O1A	0.35335 (19)	0.31206 (17)	0.27744 (14)	0.0169 (6)	
O1B	0.08759 (19)	0.32183 (18)	0.40303 (14)	0.0188 (6)	
O2B	−0.0900 (2)	0.35721 (17)	0.41491 (15)	0.0223 (6)	
H2B	−0.0909	0.3919	0.3805	0.034*	
O2A	0.1747 (2)	0.3367 (2)	0.26572 (16)	0.0290 (7)	
H2A	0.1429	0.3446	0.3028	0.043*	
O22B	−0.0711 (3)	0.2367 (2)	0.80910 (16)	0.0368 (8)	
O21B	−0.0793 (3)	0.13604 (19)	0.72486 (16)	0.0353 (8)	
O22A	0.1994 (3)	0.1316 (2)	−0.04698 (17)	0.0341 (7)	
C20A	0.4687 (3)	0.4126 (2)	0.2155 (2)	0.0157 (8)	
H20A	0.4941	0.4337	0.2585	0.019*	
N1A	0.2735 (2)	0.2432 (2)	0.15006 (17)	0.0156 (7)	
N1B	−0.0003 (2)	0.2559 (2)	0.52882 (17)	0.0157 (6)	
N2B	−0.0921 (3)	0.2144 (2)	0.74825 (18)	0.0230 (8)	
C16A	0.3515 (3)	0.3115 (2)	0.1502 (2)	0.0142 (7)	
C8A	0.2519 (3)	0.2689 (3)	0.2753 (2)	0.0168 (8)	
H8A	0.2393	0.2368	0.3201	0.02*	
C10A	0.3123 (3)	0.1121 (2)	0.23395 (18)	0.0151 (8)	
C4A	0.0388 (3)	0.4109 (3)	0.0605 (2)	0.0281 (10)	
H4A	0.0101	0.4577	0.0886	0.034*	
C5B	−0.2489 (3)	0.4199 (3)	0.6959 (2)	0.0275 (9)	
H5B	−0.294	0.4629	0.7168	0.033*	
N2A	0.1720 (3)	0.2069 (2)	−0.06923 (18)	0.0265 (8)	
C13A	0.4302 (3)	−0.0451 (3)	0.2722 (2)	0.0223 (9)	
H13A	0.4693	−0.0974	0.2849	0.027*	
C14A	0.4782 (3)	0.0306 (3)	0.2398 (2)	0.0222 (9)	
H14A	0.55	0.0292	0.2309	0.027*	
C18A	0.4715 (3)	0.4166 (2)	0.0898 (2)	0.0173 (8)	
H18A	0.4984	0.4405	0.048	0.021*	
C19B	0.2465 (3)	0.4503 (3)	0.5305 (2)	0.0242 (9)	
H19B	0.302	0.4927	0.5306	0.029*	
C6A	0.0553 (3)	0.3375 (3)	−0.0502 (2)	0.0279 (10)	
H6A	0.0382	0.3337	−0.0977	0.033*	
C1A	0.2318 (3)	0.2083 (2)	0.0850 (2)	0.0159 (8)	
H1A1	0.1954	0.1499	0.0939	0.019*	
H1A2	0.2894	0.1953	0.0531	0.019*	
C13B	0.1290 (3)	−0.0456 (3)	0.4143 (2)	0.0211 (9)	
H13B	0.1623	−0.1022	0.4048	0.025*	
C10B	0.0285 (3)	0.1225 (2)	0.44585 (19)	0.0143 (7)	
C18B	0.2053 (3)	0.4181 (3)	0.5926 (2)	0.0231 (9)	
H18B	0.2331	0.4392	0.6349	0.028*	
C9A	0.2468 (3)	0.1974 (2)	0.21582 (19)	0.0148 (8)	
H9A	0.1736	0.1766	0.2121	0.018*	
C8B	−0.0167 (3)	0.2854 (2)	0.4040 (2)	0.0184 (8)	
H8B	−0.0311	0.2556	0.3585	0.022*	
C20B	0.2038 (3)	0.4185 (2)	0.4669 (2)	0.0190 (8)	
H20B	0.2302	0.4405	0.4244	0.023*	
C21A	0.3903 (3)	0.3462 (2)	0.21365 (19)	0.0138 (8)	
C15A	0.4194 (3)	0.1085 (3)	0.2206 (2)	0.0197 (8)	
H15A	0.4522	0.1588	0.1986	0.024*	
O21A	0.1778 (3)	0.2291 (3)	−0.13132 (18)	0.0549 (11)	
C12A	0.3238 (3)	−0.0421 (3)	0.2855 (2)	0.0253 (9)	
H12A	0.2911	−0.0926	0.3073	0.03*	
C12B	0.0268 (3)	−0.0320 (3)	0.39325 (19)	0.0186 (8)	
H12B	−0.0085	−0.0787	0.3686	0.022*	
C17A	0.3930 (3)	0.3493 (2)	0.0882 (2)	0.0161 (8)	
H17A	0.3675	0.3288	0.045	0.019*	
C16B	0.0804 (3)	0.3195 (2)	0.5305 (2)	0.0150 (8)	
C6B	−0.2018 (3)	0.3512 (3)	0.7354 (2)	0.0246 (9)	
H6B	−0.213	0.3481	0.7837	0.03*	
C7B	−0.1375 (3)	0.2867 (3)	0.7026 (2)	0.0180 (8)	
C3A	0.1106 (3)	0.3480 (3)	0.0891 (2)	0.0203 (8)	
H3A	0.1288	0.3538	0.1363	0.024*	
C1B	−0.0440 (3)	0.2183 (2)	0.5937 (2)	0.0184 (8)	
H1B1	0.013	0.2008	0.6249	0.022*	
H1B2	−0.0838	0.1621	0.583	0.022*	
C17B	0.1231 (3)	0.3546 (3)	0.5934 (2)	0.0190 (8)	
H17B	0.0956	0.3349	0.6363	0.023*	
C19A	0.5096 (3)	0.4480 (2)	0.1537 (2)	0.0196 (8)	
H19A	0.5626	0.4928	0.1551	0.023*	
C2B	−0.1151 (3)	0.2880 (2)	0.6311 (2)	0.0149 (8)	
C7A	0.1272 (3)	0.2745 (3)	−0.0214 (2)	0.0193 (8)	
C5A	0.0100 (3)	0.4046 (3)	−0.0091 (2)	0.0309 (10)	
H5A	−0.0396	0.4454	−0.0279	0.037*	
C4B	−0.2285 (3)	0.4243 (3)	0.6249 (2)	0.0237 (9)	
H4B	−0.2596	0.4709	0.5978	0.028*	
C2A	0.1559 (3)	0.2770 (2)	0.0497 (2)	0.0157 (8)	
C15B	0.1323 (3)	0.1074 (3)	0.4654 (2)	0.0201 (8)	
H15B	0.1686	0.1539	0.4896	0.024*	
C21B	0.1224 (3)	0.3544 (3)	0.4673 (2)	0.0166 (8)	
C11A	0.2657 (3)	0.0354 (3)	0.2666 (2)	0.0199 (8)	
H11A	0.194	0.0365	0.2759	0.024*	
C3B	−0.1625 (3)	0.3602 (3)	0.5934 (2)	0.0199 (8)	
H3B	−0.1491	0.3654	0.5454	0.024*	
C14B	0.1824 (3)	0.0243 (3)	0.4494 (2)	0.0228 (9)	
H14B	0.2521	0.0154	0.4624	0.027*	
C9B	−0.0301 (3)	0.2126 (3)	0.46218 (19)	0.0163 (8)	
H9B	−0.1051	0.1973	0.465	0.02*	
C11B	−0.0240 (3)	0.0526 (2)	0.40908 (19)	0.0177 (8)	
H11B	−0.0931	0.0619	0.3949	0.021*	

### Atomic displacement parameters (Å^2^)

**Table:**

	*U*^11^	*U*^22^	*U*^33^	*U*^12^	*U*^13^	*U*^23^
O1A	0.0165 (14)	0.0217 (13)	0.0126 (13)	−0.0024 (10)	−0.0029 (11)	−0.0010 (11)
O1B	0.0179 (14)	0.0223 (13)	0.0162 (14)	−0.0010 (11)	−0.0002 (11)	−0.0038 (10)
O2B	0.0200 (15)	0.0231 (14)	0.0240 (15)	0.0070 (11)	−0.0034 (12)	0.0018 (11)
O2A	0.0266 (16)	0.0328 (15)	0.0277 (17)	0.0095 (13)	0.0048 (13)	0.0000 (13)
O22B	0.065 (2)	0.0267 (16)	0.0185 (18)	0.0054 (15)	−0.0077 (15)	−0.0012 (13)
O21B	0.063 (2)	0.0168 (15)	0.0258 (17)	0.0039 (13)	0.0028 (16)	−0.0007 (12)
O22A	0.049 (2)	0.0251 (16)	0.0287 (17)	0.0089 (14)	−0.0047 (15)	−0.0047 (13)
C20A	0.0142 (18)	0.0158 (17)	0.0170 (19)	0.0008 (15)	−0.0041 (15)	−0.0056 (15)
N1A	0.0174 (16)	0.0162 (15)	0.0132 (16)	−0.0016 (13)	−0.0005 (13)	0.0023 (12)
N1B	0.0185 (16)	0.0180 (15)	0.0107 (16)	−0.0047 (13)	−0.0003 (13)	−0.0038 (13)
N2B	0.029 (2)	0.0204 (18)	0.0195 (19)	−0.0011 (14)	0.0045 (15)	0.0010 (14)
C16A	0.0096 (18)	0.0136 (17)	0.020 (2)	0.0007 (14)	−0.0004 (15)	−0.0009 (14)
C8A	0.0119 (17)	0.0217 (18)	0.017 (2)	0.0025 (14)	0.0003 (15)	0.0050 (15)
C10A	0.0170 (19)	0.0183 (18)	0.0100 (18)	0.0005 (15)	0.0015 (15)	−0.0036 (14)
C4A	0.024 (2)	0.028 (2)	0.032 (2)	0.0089 (19)	0.0022 (18)	−0.0013 (18)
C5B	0.021 (2)	0.029 (2)	0.033 (2)	0.0070 (18)	0.0082 (18)	−0.0042 (18)
N2A	0.036 (2)	0.0277 (19)	0.016 (2)	0.0025 (16)	−0.0012 (16)	0.0003 (14)
C13A	0.030 (2)	0.0200 (19)	0.017 (2)	0.0072 (16)	0.0006 (17)	−0.0013 (15)
C14A	0.0176 (19)	0.0186 (19)	0.030 (2)	0.0043 (15)	0.0035 (17)	−0.0020 (16)
C18A	0.0161 (19)	0.0146 (18)	0.021 (2)	0.0003 (16)	0.0007 (16)	0.0043 (15)
C19B	0.0126 (19)	0.0184 (19)	0.042 (3)	−0.0017 (15)	−0.0027 (18)	−0.0051 (18)
C6A	0.031 (2)	0.035 (2)	0.017 (2)	−0.0023 (19)	−0.0083 (19)	0.0076 (17)
C1A	0.0109 (18)	0.0176 (19)	0.019 (2)	−0.0025 (14)	−0.0031 (16)	−0.0023 (15)
C13B	0.033 (2)	0.0149 (18)	0.0155 (19)	0.0057 (16)	0.0098 (18)	−0.0005 (14)
C10B	0.0158 (18)	0.0165 (17)	0.0107 (18)	−0.0038 (14)	0.0017 (15)	0.0011 (14)
C18B	0.018 (2)	0.021 (2)	0.031 (2)	0.0032 (17)	−0.0048 (18)	−0.0104 (17)
C9A	0.0119 (18)	0.0184 (18)	0.0140 (19)	−0.0021 (14)	0.0011 (15)	0.0037 (14)
C8B	0.0165 (19)	0.0211 (19)	0.018 (2)	0.0013 (15)	−0.0035 (16)	−0.0037 (15)
C20B	0.0143 (19)	0.0160 (19)	0.027 (2)	0.0021 (15)	0.0031 (16)	0.0010 (16)
C21A	0.0116 (17)	0.0175 (19)	0.0122 (19)	0.0048 (14)	−0.0011 (15)	0.0021 (14)
C15A	0.019 (2)	0.0181 (19)	0.022 (2)	0.0004 (15)	0.0054 (17)	0.0004 (16)
O21A	0.085 (3)	0.058 (2)	0.022 (2)	0.031 (2)	0.0133 (19)	0.0036 (16)
C12A	0.030 (2)	0.0213 (19)	0.024 (2)	−0.0002 (17)	0.0033 (19)	0.0067 (17)
C12B	0.025 (2)	0.0182 (18)	0.0129 (19)	−0.0037 (15)	0.0028 (16)	−0.0066 (14)
C17A	0.0142 (19)	0.0178 (19)	0.0162 (19)	0.0020 (15)	−0.0026 (16)	0.0007 (15)
C16B	0.0121 (18)	0.0182 (18)	0.0148 (19)	0.0053 (15)	−0.0020 (15)	−0.0042 (14)
C6B	0.026 (2)	0.031 (2)	0.017 (2)	−0.0018 (18)	0.0058 (17)	0.0006 (16)
C7B	0.0161 (19)	0.017 (2)	0.021 (2)	−0.0041 (15)	0.0012 (16)	0.0028 (15)
C3A	0.019 (2)	0.023 (2)	0.020 (2)	0.0020 (16)	−0.0004 (17)	−0.0026 (16)
C1B	0.021 (2)	0.0147 (18)	0.020 (2)	−0.0007 (16)	−0.0014 (17)	−0.0009 (15)
C17B	0.018 (2)	0.021 (2)	0.018 (2)	0.0023 (16)	−0.0016 (16)	−0.0041 (15)
C19A	0.0130 (18)	0.0157 (18)	0.030 (2)	−0.0009 (15)	−0.0030 (17)	0.0003 (16)
C2B	0.0112 (18)	0.0133 (17)	0.020 (2)	−0.0040 (14)	0.0000 (15)	−0.0005 (14)
C7A	0.022 (2)	0.020 (2)	0.015 (2)	−0.0036 (15)	−0.0007 (16)	0.0013 (15)
C5A	0.024 (2)	0.035 (2)	0.034 (3)	0.013 (2)	−0.0023 (19)	0.0071 (19)
C4B	0.017 (2)	0.024 (2)	0.030 (2)	0.0067 (17)	−0.0015 (17)	0.0034 (17)
C2A	0.0142 (18)	0.0144 (18)	0.019 (2)	−0.0053 (15)	−0.0010 (15)	0.0039 (14)
C15B	0.018 (2)	0.020 (2)	0.022 (2)	0.0006 (16)	−0.0007 (16)	−0.0030 (16)
C21B	0.015 (2)	0.0167 (19)	0.018 (2)	0.0022 (15)	0.0002 (16)	−0.0049 (15)
C11A	0.015 (2)	0.027 (2)	0.017 (2)	−0.0021 (16)	0.0008 (15)	0.0062 (15)
C3B	0.018 (2)	0.023 (2)	0.018 (2)	0.0012 (16)	−0.0009 (16)	0.0009 (16)
C14B	0.018 (2)	0.027 (2)	0.022 (2)	0.0055 (16)	−0.0006 (17)	0.0008 (17)
C9B	0.0094 (18)	0.025 (2)	0.014 (2)	−0.0016 (15)	−0.0006 (15)	−0.0039 (15)
C11B	0.0180 (19)	0.0220 (19)	0.0130 (19)	−0.0021 (15)	0.0006 (16)	−0.0012 (15)

### Geometric parameters (Å, º)

**Table:**

O1A—C21A	1.389 (4)	C6A—C7A	1.395 (6)
O1A—C8A	1.432 (4)	C6A—H6A	0.93
O1B—C21B	1.379 (5)	C1A—C2A	1.532 (5)
O1B—C8B	1.426 (4)	C1A—H1A1	0.97
O2B—C8B	1.402 (4)	C1A—H1A2	0.97
O2B—H2B	0.82	C13B—C12B	1.375 (6)
O2A—C8A	1.391 (4)	C13B—C14B	1.380 (6)
O2A—H2A	0.82	C13B—H13B	0.93
O22B—N2B	1.229 (5)	C10B—C11B	1.390 (5)
O21B—N2B	1.215 (4)	C10B—C15B	1.390 (5)
O22A—N2A	1.207 (4)	C10B—C9B	1.519 (5)
C20A—C21A	1.376 (5)	C18B—C17B	1.385 (6)
C20A—C19A	1.380 (6)	C18B—H18B	0.93
C20A—H20A	0.93	C9A—H9A	0.98
N1A—C16A	1.393 (4)	C8B—C9B	1.527 (5)
N1A—C1A	1.434 (5)	C8B—H8B	0.98
N1A—C9A	1.451 (5)	C20B—C21B	1.382 (5)
N1B—C16B	1.372 (5)	C20B—H20B	0.93
N1B—C1B	1.455 (5)	C15A—H15A	0.93
N1B—C9B	1.459 (5)	C12A—C11A	1.379 (5)
N2B—C7B	1.467 (5)	C12A—H12A	0.93
C16A—C21A	1.393 (5)	C12B—C11B	1.403 (5)
C16A—C17A	1.401 (5)	C12B—H12B	0.93
C8A—C9A	1.524 (5)	C17A—H17A	0.93
C8A—H8A	0.98	C16B—C17B	1.406 (5)
C10A—C15A	1.389 (5)	C16B—C21B	1.407 (5)
C10A—C11A	1.392 (5)	C6B—C7B	1.381 (6)
C10A—C9A	1.516 (5)	C6B—H6B	0.93
C4A—C5A	1.375 (6)	C7B—C2B	1.389 (6)
C4A—C3A	1.393 (6)	C3A—C2A	1.386 (5)
C4A—H4A	0.93	C3A—H3A	0.93
C5B—C6B	1.375 (6)	C1B—C2B	1.522 (5)
C5B—C4B	1.376 (6)	C1B—H1B1	0.97
C5B—H5B	0.93	C1B—H1B2	0.97
N2A—O21A	1.224 (5)	C17B—H17B	0.93
N2A—C7A	1.443 (5)	C19A—H19A	0.93
C13A—C12A	1.378 (6)	C2B—C3B	1.393 (5)
C13A—C14A	1.386 (5)	C7A—C2A	1.401 (6)
C13A—H13A	0.93	C5A—H5A	0.93
C14A—C15A	1.390 (5)	C4B—C3B	1.378 (6)
C14A—H14A	0.93	C4B—H4B	0.93
C18A—C19A	1.384 (6)	C15B—C14B	1.381 (5)
C18A—C17A	1.386 (5)	C15B—H15B	0.93
C18A—H18A	0.93	C11A—H11A	0.93
C19B—C18B	1.370 (6)	C3B—H3B	0.93
C19B—C20B	1.401 (6)	C14B—H14B	0.93
C19B—H19B	0.93	C9B—H9B	0.98
C6A—C5A	1.363 (6)	C11B—H11B	0.93
			
C21A—O1A—C8A	115.6 (3)	C21B—C20B—H20B	119.9
C21B—O1B—C8B	114.3 (3)	C19B—C20B—H20B	119.9
C8B—O2B—H2B	109.5	C20A—C21A—O1A	117.8 (3)
C8A—O2A—H2A	109.5	C20A—C21A—C16A	121.6 (3)
C21A—C20A—C19A	120.3 (3)	O1A—C21A—C16A	120.6 (3)
C21A—C20A—H20A	119.8	C10A—C15A—C14A	120.7 (4)
C19A—C20A—H20A	119.8	C10A—C15A—H15A	119.6
C16A—N1A—C1A	120.6 (3)	C14A—C15A—H15A	119.6
C16A—N1A—C9A	118.7 (3)	C13A—C12A—C11A	120.3 (4)
C1A—N1A—C9A	119.9 (3)	C13A—C12A—H12A	119.9
C16B—N1B—C1B	120.7 (3)	C11A—C12A—H12A	119.9
C16B—N1B—C9B	119.7 (3)	C13B—C12B—C11B	119.7 (3)
C1B—N1B—C9B	118.6 (3)	C13B—C12B—H12B	120.2
O21B—N2B—O22B	123.6 (4)	C11B—C12B—H12B	120.2
O21B—N2B—C7B	119.0 (3)	C18A—C17A—C16A	121.4 (4)
O22B—N2B—C7B	117.4 (3)	C18A—C17A—H17A	119.3
N1A—C16A—C21A	120.3 (3)	C16A—C17A—H17A	119.3
N1A—C16A—C17A	122.5 (3)	N1B—C16B—C17B	123.1 (3)
C21A—C16A—C17A	117.2 (3)	N1B—C16B—C21B	120.0 (3)
O2A—C8A—O1A	110.0 (3)	C17B—C16B—C21B	116.9 (3)
O2A—C8A—C9A	109.8 (3)	C5B—C6B—C7B	119.2 (4)
O1A—C8A—C9A	110.4 (3)	C5B—C6B—H6B	120.4
O2A—C8A—H8A	108.9	C7B—C6B—H6B	120.4
O1A—C8A—H8A	108.9	C6B—C7B—C2B	123.7 (4)
C9A—C8A—H8A	108.9	C6B—C7B—N2B	115.8 (3)
C15A—C10A—C11A	118.1 (3)	C2B—C7B—N2B	120.5 (3)
C15A—C10A—C9A	122.0 (3)	C2A—C3A—C4A	122.2 (4)
C11A—C10A—C9A	119.9 (3)	C2A—C3A—H3A	118.9
C5A—C4A—C3A	120.6 (4)	C4A—C3A—H3A	118.9
C5A—C4A—H4A	119.7	N1B—C1B—C2B	112.4 (3)
C3A—C4A—H4A	119.7	N1B—C1B—H1B1	109.1
C6B—C5B—C4B	119.1 (4)	C2B—C1B—H1B1	109.1
C6B—C5B—H5B	120.5	N1B—C1B—H1B2	109.1
C4B—C5B—H5B	120.5	C2B—C1B—H1B2	109.1
O22A—N2A—O21A	123.4 (4)	H1B1—C1B—H1B2	107.9
O22A—N2A—C7A	119.3 (3)	C18B—C17B—C16B	121.1 (4)
O21A—N2A—C7A	117.2 (3)	C18B—C17B—H17B	119.5
C12A—C13A—C14A	119.4 (4)	C16B—C17B—H17B	119.5
C12A—C13A—H13A	120.3	C20A—C19A—C18A	119.7 (3)
C14A—C13A—H13A	120.3	C20A—C19A—H19A	120.2
C13A—C14A—C15A	120.2 (4)	C18A—C19A—H19A	120.2
C13A—C14A—H14A	119.9	C7B—C2B—C3B	115.1 (3)
C15A—C14A—H14A	119.9	C7B—C2B—C1B	124.7 (3)
C19A—C18A—C17A	119.8 (4)	C3B—C2B—C1B	120.1 (4)
C19A—C18A—H18A	120.1	C6A—C7A—C2A	122.2 (4)
C17A—C18A—H18A	120.1	C6A—C7A—N2A	116.4 (4)
C18B—C19B—C20B	119.0 (4)	C2A—C7A—N2A	121.4 (3)
C18B—C19B—H19B	120.5	C6A—C5A—C4A	118.9 (4)
C20B—C19B—H19B	120.5	C6A—C5A—H5A	120.6
C5A—C6A—C7A	120.5 (4)	C4A—C5A—H5A	120.6
C5A—C6A—H6A	119.8	C5B—C4B—C3B	120.7 (4)
C7A—C6A—H6A	119.8	C5B—C4B—H4B	119.6
N1A—C1A—C2A	112.9 (3)	C3B—C4B—H4B	119.6
N1A—C1A—H1A1	109	C3A—C2A—C7A	115.6 (4)
C2A—C1A—H1A1	109	C3A—C2A—C1A	119.6 (3)
N1A—C1A—H1A2	109	C7A—C2A—C1A	124.8 (3)
C2A—C1A—H1A2	109	C14B—C15B—C10B	120.9 (4)
H1A1—C1A—H1A2	107.8	C14B—C15B—H15B	119.5
C12B—C13B—C14B	120.3 (3)	C10B—C15B—H15B	119.5
C12B—C13B—H13B	119.8	O1B—C21B—C20B	117.4 (3)
C14B—C13B—H13B	119.8	O1B—C21B—C16B	121.0 (3)
C11B—C10B—C15B	118.7 (3)	C20B—C21B—C16B	121.6 (4)
C11B—C10B—C9B	118.3 (3)	C12A—C11A—C10A	121.3 (4)
C15B—C10B—C9B	123.0 (3)	C12A—C11A—H11A	119.4
C19B—C18B—C17B	121.3 (4)	C10A—C11A—H11A	119.4
C19B—C18B—H18B	119.4	C4B—C3B—C2B	122.1 (4)
C17B—C18B—H18B	119.4	C4B—C3B—H3B	118.9
N1A—C9A—C10A	115.4 (3)	C2B—C3B—H3B	118.9
N1A—C9A—C8A	109.0 (3)	C13B—C14B—C15B	120.0 (4)
C10A—C9A—C8A	110.3 (3)	C13B—C14B—H14B	120
N1A—C9A—H9A	107.3	C15B—C14B—H14B	120
C10A—C9A—H9A	107.3	N1B—C9B—C10B	114.1 (3)
C8A—C9A—H9A	107.3	N1B—C9B—C8B	108.1 (3)
O2B—C8B—O1B	110.8 (3)	C10B—C9B—C8B	111.9 (3)
O2B—C8B—C9B	108.4 (3)	N1B—C9B—H9B	107.5
O1B—C8B—C9B	111.2 (3)	C10B—C9B—H9B	107.5
O2B—C8B—H8B	108.8	C8B—C9B—H9B	107.5
O1B—C8B—H8B	108.8	C10B—C11B—C12B	120.3 (3)
C9B—C8B—H8B	108.8	C10B—C11B—H11B	119.8
C21B—C20B—C19B	120.1 (4)	C12B—C11B—H11B	119.8
			
C1A—N1A—C16A—C21A	178.9 (3)	C21A—C20A—C19A—C18A	0.1 (5)
C9A—N1A—C16A—C21A	−10.7 (5)	C17A—C18A—C19A—C20A	−0.5 (5)
C1A—N1A—C16A—C17A	−0.1 (5)	C6B—C7B—C2B—C3B	0.0 (5)
C9A—N1A—C16A—C17A	170.3 (3)	N2B—C7B—C2B—C3B	−179.8 (3)
C21A—O1A—C8A—O2A	−71.7 (4)	C6B—C7B—C2B—C1B	179.6 (4)
C21A—O1A—C8A—C9A	49.7 (4)	N2B—C7B—C2B—C1B	−0.2 (6)
C12A—C13A—C14A—C15A	−0.3 (6)	N1B—C1B—C2B—C7B	−156.3 (3)
C16A—N1A—C1A—C2A	−75.7 (4)	N1B—C1B—C2B—C3B	23.2 (5)
C9A—N1A—C1A—C2A	114.0 (3)	C5A—C6A—C7A—C2A	0.0 (6)
C20B—C19B—C18B—C17B	0.3 (6)	C5A—C6A—C7A—N2A	−179.2 (4)
C16A—N1A—C9A—C10A	−87.3 (4)	O22A—N2A—C7A—C6A	−151.1 (4)
C1A—N1A—C9A—C10A	83.1 (4)	O21A—N2A—C7A—C6A	27.3 (6)
C16A—N1A—C9A—C8A	37.4 (4)	O22A—N2A—C7A—C2A	29.7 (6)
C1A—N1A—C9A—C8A	−152.2 (3)	O21A—N2A—C7A—C2A	−151.9 (4)
C15A—C10A—C9A—N1A	38.8 (5)	C7A—C6A—C5A—C4A	2.0 (7)
C11A—C10A—C9A—N1A	−142.9 (3)	C3A—C4A—C5A—C6A	−2.1 (7)
C15A—C10A—C9A—C8A	−85.3 (4)	C6B—C5B—C4B—C3B	0.6 (7)
C11A—C10A—C9A—C8A	93.0 (4)	C4A—C3A—C2A—C7A	1.9 (6)
O2A—C8A—C9A—N1A	65.3 (4)	C4A—C3A—C2A—C1A	−178.2 (4)
O1A—C8A—C9A—N1A	−56.1 (4)	C6A—C7A—C2A—C3A	−1.9 (6)
O2A—C8A—C9A—C10A	−167.0 (3)	N2A—C7A—C2A—C3A	177.3 (3)
O1A—C8A—C9A—C10A	71.5 (4)	C6A—C7A—C2A—C1A	178.1 (4)
C21B—O1B—C8B—O2B	68.7 (4)	N2A—C7A—C2A—C1A	−2.7 (6)
C21B—O1B—C8B—C9B	−51.9 (4)	N1A—C1A—C2A—C3A	−19.0 (5)
C18B—C19B—C20B—C21B	−1.0 (5)	N1A—C1A—C2A—C7A	161.0 (3)
C19A—C20A—C21A—O1A	179.0 (3)	C11B—C10B—C15B—C14B	−0.7 (6)
C19A—C20A—C21A—C16A	1.3 (5)	C9B—C10B—C15B—C14B	180.0 (4)
C8A—O1A—C21A—C20A	159.9 (3)	C8B—O1B—C21B—C20B	−158.0 (3)
C8A—O1A—C21A—C16A	−22.3 (4)	C8B—O1B—C21B—C16B	25.5 (5)
N1A—C16A—C21A—C20A	178.9 (3)	C19B—C20B—C21B—O1B	−176.5 (3)
C17A—C16A—C21A—C20A	−2.1 (5)	C19B—C20B—C21B—C16B	0.0 (5)
N1A—C16A—C21A—O1A	1.2 (5)	N1B—C16B—C21B—O1B	−3.5 (5)
C17A—C16A—C21A—O1A	−179.7 (3)	C17B—C16B—C21B—O1B	178.0 (3)
C11A—C10A—C15A—C14A	−0.4 (6)	N1B—C16B—C21B—C20B	−179.9 (3)
C9A—C10A—C15A—C14A	178.0 (3)	C17B—C16B—C21B—C20B	1.6 (5)
C13A—C14A—C15A—C10A	0.4 (6)	C13A—C12A—C11A—C10A	0.0 (6)
C14A—C13A—C12A—C11A	0.1 (6)	C15A—C10A—C11A—C12A	0.2 (6)
C14B—C13B—C12B—C11B	−1.4 (6)	C9A—C10A—C11A—C12A	−178.2 (4)
C19A—C18A—C17A—C16A	−0.4 (5)	C5B—C4B—C3B—C2B	1.0 (6)
N1A—C16A—C17A—C18A	−179.3 (3)	C7B—C2B—C3B—C4B	−1.3 (5)
C21A—C16A—C17A—C18A	1.7 (5)	C1B—C2B—C3B—C4B	179.1 (4)
C1B—N1B—C16B—C17B	−2.3 (5)	C12B—C13B—C14B—C15B	1.8 (6)
C9B—N1B—C16B—C17B	−171.1 (3)	C10B—C15B—C14B—C13B	−0.8 (6)
C1B—N1B—C16B—C21B	179.3 (3)	C16B—N1B—C9B—C10B	89.6 (4)
C9B—N1B—C16B—C21B	10.4 (5)	C1B—N1B—C9B—C10B	−79.4 (4)
C4B—C5B—C6B—C7B	−1.8 (6)	C16B—N1B—C9B—C8B	−35.5 (4)
C5B—C6B—C7B—C2B	1.5 (6)	C1B—N1B—C9B—C8B	155.4 (3)
C5B—C6B—C7B—N2B	−178.7 (4)	C11B—C10B—C9B—N1B	146.7 (3)
O21B—N2B—C7B—C6B	145.4 (4)	C15B—C10B—C9B—N1B	−33.9 (5)
O22B—N2B—C7B—C6B	−33.2 (5)	C11B—C10B—C9B—C8B	−90.2 (4)
O21B—N2B—C7B—C2B	−34.8 (5)	C15B—C10B—C9B—C8B	89.2 (4)
O22B—N2B—C7B—C2B	146.6 (4)	O2B—C8B—C9B—N1B	−66.3 (4)
C5A—C4A—C3A—C2A	0.0 (7)	O1B—C8B—C9B—N1B	55.8 (4)
C16B—N1B—C1B—C2B	75.9 (4)	O2B—C8B—C9B—C10B	167.3 (3)
C9B—N1B—C1B—C2B	−115.1 (3)	O1B—C8B—C9B—C10B	−70.7 (4)
C19B—C18B—C17B—C16B	1.4 (6)	C15B—C10B—C11B—C12B	1.0 (5)
N1B—C16B—C17B—C18B	179.3 (3)	C9B—C10B—C11B—C12B	−179.5 (3)
C21B—C16B—C17B—C18B	−2.3 (5)	C13B—C12B—C11B—C10B	0.0 (5)

### Hydrogen-bond geometry (Å, º)

Cg1 and Cg2 are the controids of the C10A–C15A and C10B–C15B rings, respectively.

**Table:**

*D*—H···*A*	*D*—H	H···*A*	*D*···*A*	*D*—H···*A*
O2*A*—H2*A*···O1*B*	0.82	2.06	2.843 (4)	161
C6*B*—H6*B*···O21*A*^i^	0.93	2.40	3.175 (5)	141
C8*B*—H8*B*···O1*A*^ii^	0.98	2.34	3.234 (4)	151
C14*A*—H14*A*···O2*A*^iii^	0.93	2.57	3.177 (5)	123
C19*A*—H19*A*···O21*B*^iv^	0.93	2.45	3.134 (4)	131
C19*B*—H19*B*···O22*A*^v^	0.93	2.47	3.057 (5)	121
O2*B*—H2*B*···*Cg*1^ii^	0.82	2.69	3.484 (3)	164
C18*A*—H18*A*···*Cg*2^iv^	0.93	2.83	3.564 (4)	137
C18*B*—H18*B*···*Cg*1^v^	0.93	2.94	3.601 (4)	130

Symmetry codes: (i) *x*−1/2, −*y*+1/2, *z*+1; (ii) *x*−1/2, −*y*+1/2, *z*; (iii) *x*+1/2, −*y*+1/2, *z*; (iv) −*x*+1/2, *y*+1/2, *z*−1/2; (v) −*x*+1/2, *y*+1/2, *z*+1/2.
